# Unusual Presentation of a Giant Cell Tumor of the Bone: A Case Report

**DOI:** 10.7759/cureus.56929

**Published:** 2024-03-25

**Authors:** Prachi Gedekar, Atul Chavhan, K. M. Hiwale, Shakti Sagar

**Affiliations:** 1 Pathology, Datta Meghe Institute of Medical Sciences, Datta Meghe Institute of Higher Education and Research, Wardha, IND

**Keywords:** bone malignancies, histopathology, tibia, bone tissue, giant cell tumor

## Abstract

Bone giant cell tumors (GCTs) are rare, non-cancerous tumors that mostly affect the meta-epiphyseal region of long bones in the legs and arms. We are reporting a case of GCT of bone of a 14-year-old male; it usually occurs in the age group of 20-40 years. The presence of multinucleated giant cells and stromal cells in the proximal diaphysis of the left tibia serves as a distinguishing characteristic. The majority of GCTs are benign; they have the potential to induce bone loss and can be locally aggressive. Treatment options often include surgery, and in some cases, medications like denosumab may be used to help shrink the tumor or manage recurrent cases.

## Introduction

The rare benign primary bone tumor, a giant cell tumor of bone (GCTB), comprises a reactive state and spindle-shaped neoplastic cells. The receptor activator of nuclear factor-kappa B (RANK) makes up this structure [1].

A giant cell tumor (GCT) is a type of malignancy that primarily attacks the bone locally and seldom spreads to other sites. GCTB can be detected before surgery when the right radiographic data are obtained [2]. Furthermore, given the aggressive character of the tumor and its rare tendency to progress malignantly, an intraoperative frozen section or core needle biopsy is recommended to confirm the diagnosis, either before or during surgery [3]. GCT is commonly referred to as osteoblastoma because of its abnormally high population of multinucleated giant cells [4]. GCTB has an uncommon propensity to spread, and malignant tumors have several features such as uncontrolled growth, invasion, metastasis, abnormal cell structure, angiogenesis, and destructive growth [5].

Although they can be locally aggressive, GCTBs are generally thought to be benign [6]. The GCTB is a non-cancerous osteolytic tumor characterized by three primary cellular components, including multinucleated giant cells resembling osteoclasts, stromal cells with spindle-like morphology, and single nucleus cells belonging to the monocyte/macrophage lineage [7]. Primary treatment encompasses surgery, radiotherapy, and systemic interventions [8].

The typical treatment for GCTB involves surgically removing the tumor either through curettage or resections. In some cases, resection may not be feasible at some sites, even with intraoperative adjuvant therapy (e.g., skull, spine) [9].

Pathogenic sickness and evolutionary dynamics are significantly impacted by cellular mechanisms, particularly those related to proinflammatory and immunological responses, as well as depression [10]. GCTB is an uncommon stromal tumor associated with osteoclast formation. A rare occurrence in GCTs of the bone is malignant transformation [11].

GCT of bone remains very complex and extensively researched bone tumors. The origin of GCTs in bones is still unclear [12]. The GCT of bone occurs in the lower end of the radius, the lower end of the femur, and the upper end of the tibia. They originate from the area where the epiphysis-metaphysis extends into the bone plate beneath the joint surface [13].

## Case presentation

We are reporting a case of GCT of the left tibia in a 14-year-old male who presented with a history of pain in the left foot and reduced ability to walk. The pain was dull and non-radiating, aggravated at night. Then, one day, the patient fell while playing in school and heard a cracking sound in their leg. The nature of the pain was sharp shooting with sudden onset and progression, which increased in severity. He was unable to bear weight after an incident. He consulted a local physician, and he was recommended for a non-contrast computed tomography (NCCT)/MRI as well as given analgesics.

**Figure 1:**
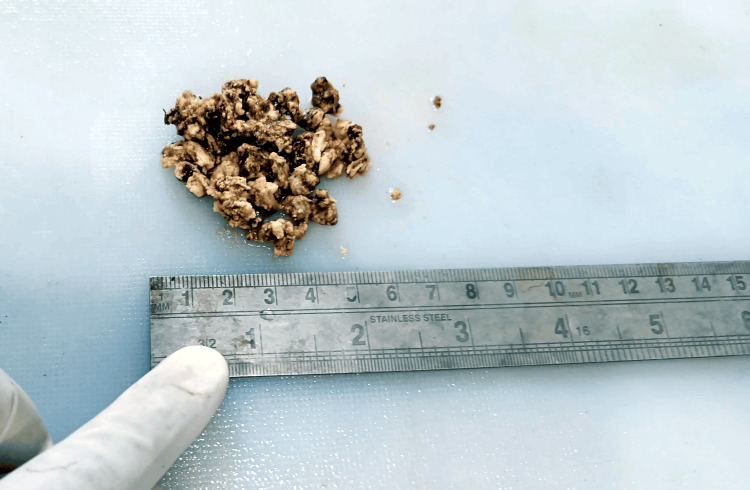
Gross specimen of giant cell tumor

The clinical examination showed swelling and tenderness. NCCT revealed a well-defined centric intramedullary lytic lesion with peripheral areas of sclerosis measuring approximately 64 × 29 × 22 mm involving the proximal diaphysis of the left tibia with adjacent cortical thinning in the medial pathological fracture in the diaphysis of the tibia that was accompanied by a displaced fracture, most likely caused by a benign neoplastic etiology. A lytic lesion with an eccentric, soap bubble appearance growth involving the metaphysis of the tibia has been observed (Figure 1). General hematology results, such as CBC, ESR, and coagulation profile, are normal. Biochemical examination shows normal liver function test (LFT), kidney function test (KFT), vitamin, and calcium.

**Figure 2:**
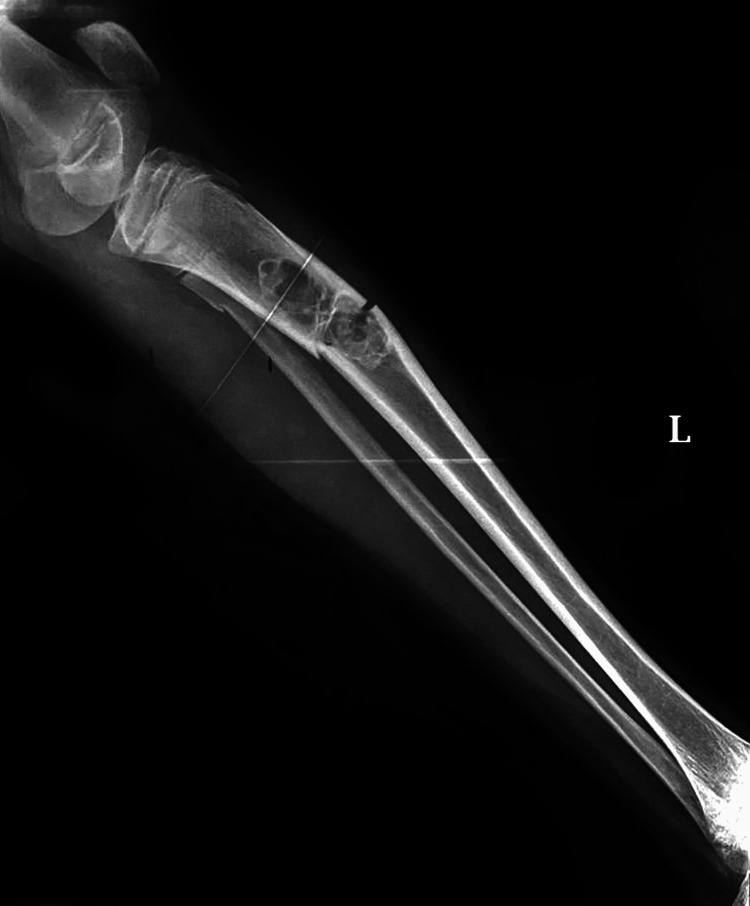
Non-contrast computed tomography scan of left leg showing lytic lesion, eccentric, and soap bubble appearance growth involving metaphysis of the tibia

**Figure 3:**
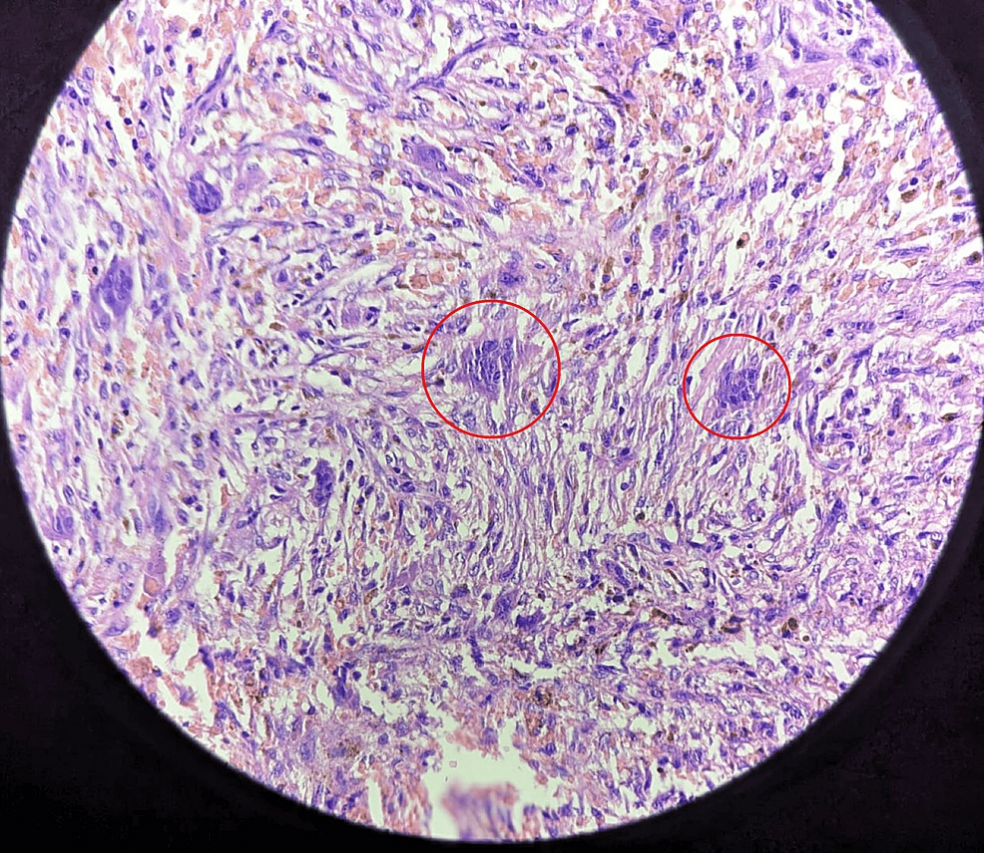
Microscopy examination of giant cell tumor showing round and oval spindle cells with multinucleated giant cells

The expertise and guidance of the tertiary level orthopedic oncology center were sought, and an orthopedic consultant specialist in foot surgery performed the procedure in consultation with the surgical specialist under spinal anesthesia, open reduction, and internal fixation with plate osteosynthesis for proximal 1/3 proximal shaft of the tibia fracture left side. This was under aseptic precaution, cleaning, painting, and draping. The procedure was completed by closing the suture site in layers as muscle and subcutaneous tissue using Ethilon 2-0 and Vicryl 1-0. He was instructed to return for re-dressing and stitch removal in one week. The treatment from postoperative instruction is shown in Table 1. Multiple samples are taken and sent for histology tests. The specimens in the four containers received are labeled as bone tissue. It consisted of multiple irregular, brownish, and bony tissue pieces aggregating 5 × 4 × 2 cm (Figure 2). Results of the histology test revealed the presence of round and oval cells and spindle cells with pale eosinophilic cytoplasm and multinucleated giant cells and hemorrhage by microscopy (Figure 3).

**Table 1:** Treatment given to the patient The medications with doses and durations given to the patient. NBM, nil by mouth; NS, normal saline

Treatment	Names
IV fluids	DNS 500 mL (90 mL/hour)
Drugs	Injection Pantoprazole 40 mg IV once daily for one day
	Tablets Pantoprazole 40 mg once daily
Pain control	Injection Paracetamol 50 mL IV twice daily for five days
	Injection Tramadol 35 mg in 50 mL NS over one hour as needed
Antibiotic	Injection Ceftriaxone and Sulbactam 1.5 g IV twice daily for five days
	Injection Amikacin 500 mg once daily for three days
Other medication	After NBM release: tab vitamin C 500 mg twice daily
	Tablets Trypsin-Chymotrypsin twice daily

The patient was called for follow-up after one month. The surgical wound healed well and suggested physiotherapy regularly for better improvement. 

## Discussion

According to Lipplaa et al., GCTB is a tumor with intermediate local aggressiveness, but metastasis is infrequent. Treatment decisions should involve a multidisciplinary team comprising specialists in musculoskeletal oncology. This team should incorporate a radiographic evaluation, a specialized MRI, a histological examination, and the scheduled operation. Additionally, targeted, comprehensive treatment may be included if deemed necessary [1].

van der Heijden et al.’s study indicates that an effective approach for tumor removal and preventing recurrence might involve a combination of therapies, such as through surgical excision complemented by other adjuvant treatments. Furthermore, molecular medications directed at cellular components implicated in the pathology of GCTs could potentially reduce recurrence rates. A contemporary emphasis on understanding the pathophysiology will likely lead to an excess of mechanism-based treatments for GCT [4].

A study by Palmerini et al. states that detecting malignant GCTs of bone is rare, and their diagnosis is difficult because there are no clearly defined diagnostic criteria available. For the possible care of individuals with bone giant tumors, we advise histologic sampling to guarantee a precise diagnosis, vigilant monitoring, especially for patients undergoing radiation therapy, and prompt intervention for any local recurrence and crucial components of follow-up care [6].

As stated in López-Pousa et al.’s study, GCTB is a primary bone tumor characterized by bone destruction and aggression. It is quite aggressive and causes a substantial amount of morbidity. GCTB comprises giant cells resembling osteoclast expressing RANK, along with stromal cells expressing RANK, a pivotal factor influencing the formation, activation, survival, and function of osteoclasts. Overproduction of RANK leads to a disruption in bone remodeling, favoring the process of bone breakdown [7].

## Conclusions

This case report included the diagnosis and course of treatment for a rare manifestation of GCT in a 14-year-old male in the left tibia proximal diaphysis. Through examination, imaging studies, and histopathological analysis, the tumor was identified and appropriately treated with surgical resection. Close monitoring post-surgery revealed no recurrence, emphasizing the importance of regular follow-up in managing such cases.

This report contributes to the existing literature on GCTs, underscoring the significance of accurate diagnosis and multidisciplinary management for optimal patient outcomes. Further research and long-term studies are warranted to enhance our understanding and treatment strategies for this rare but potentially aggressive tumor. Accurate diagnosis of atypical cancer requires knowledge of its sites.
